# A multilevel analysis of lifestyle variations in symptoms of acute respiratory infection among young children under five in Nigeria

**DOI:** 10.1186/s12889-016-3565-0

**Published:** 2016-08-25

**Authors:** Oluwafunmilade A. Adesanya, Chi Chiao

**Affiliations:** 1 Institute of Public Health, International Health Program, School of Medicine, Institute of Public Health, National Yang-Ming University, Taipei, Taiwan; 2 School of Medicine, Institute of Health and Welfare Policy, National Yang-Ming University, No. 155, Sec. 2, Li-Nong St, 112 Taipei, Taiwan People’s Republic of China

**Keywords:** (MeSH): Symptoms of acute respiratory infection (ARI), Lifestyle factors, Multilevel analysis, Young children, Nigeria

## Abstract

**Background:**

Nigeria has the second highest estimated number of deaths due to acute respiratory infection (ARI) among children under five in the world. A common hypothesis is that the inequitable distribution of socioeconomic resources shapes individual lifestyles and health behaviors, which leads to poorer health, including symptoms of ARI. This study examined whether lifestyle factors are associated with ARI risk among Nigerian children aged less than 5 years, taking individual-level and contextual-level risk factors into consideration.

**Methods:**

Data were obtained from the nationally representative 2013 Nigeria Demographic and Health Survey. A total of 28,596 surviving children aged 5 years or younger living in 896 communities were analyzed. We employed two-level multilevel logistic regressions to model the relationship between lifestyle factors and ARI symptoms.

**Results:**

The multivariate results from multilevel regressions indicated that the odds of having ARI symptoms were increased by a number of lifestyle factors such as in-house biomass cooking (OR = 2.30; *p* < 0.01) and no hand-washing (OR = 1.66; *p* < 0.001). An increased risk of ARI symptoms was also significantly associated with living in the North West region and the community with a high proportion of orphaned/vulnerable children (OR = 1.74; *p* < 0.001).

**Conclusions:**

Our findings underscore the importance of Nigerian children’s lifestyle within the neighborhoods where they reside above their individual characteristics. Program-based strategies that are aimed at reducing ARI symptoms should consider policies that embrace making available basic housing standards, providing improved cooking stoves and enhancing healthy behaviors.

## Background

Acute respiratory tract infection (ARI) remains one of major infectious causes of mortality and morbidity among children and it accounts for more than one-third of mortality among children under 5 years old; it results in approximately 2 million deaths per year around the world [1, 2]. In developing countries, 70 % of childhood morbidities among children under 5 years are due to ARI [3] with an average annual incidence of five–six episodes of ARI per child per year [4]. Sub-Saharan Africa is the second highest contributor to the global count of ARI [5, 6]. As such infections are one of major causes of pediatric outpatient visits [7], with 27 to 96 % of such visits resulting in hospitalization [8]. Prior studies have shown that the determinants of a child health are influenced by their personal lifestyle factors, namely those that are practiced within their household and community [9]. Specifically, findings from a randomized control trial and a systematic review have both confirmed that practicing hand-washing by individuals and the promotion of hand-washing among community members are able to significantly reduce the risk of ARI [10, 11].

In line with this evidence, lifestyle factors practiced in households are believed to be highly important determinants of child health outcomes. To measure lifestyle factors, Mishra and his colleagues measured the extent of exposure to cooking smoke by examining the type of fuel used for cooking in households; these were grouped into three categories (high, medium and low pollution fuels) in order to represent the extent of exposure to cooking smoke [12]. In another study, Kilabuko and Nakai, employing the same type of question, but categorizing children into two groups: was able to separate children into those from homes using biomass fuels or those from homes using kerosene/charcoal [13]. Furthermore, a population based study from India also measured the extent of exposure to cooking smoke; this was done by examining what type of fuel households uses most commonly for cooking; by examining whether household members smoked and identifying whether there was a separate kitchen [14].

Given the important influence of household practices on child health as these become integrated with various behavioral and lifestyle factors, studies that only account for one or two modifiable lifestyle practices are very likely not able to fully capture the whole range of factors that a child with ARI is exposed to. To be specific, prior studies seem to have overlooked the possibility that there is synergy between biomass based cooking and the place where cooking takes place; for example; effective exposure is likely to be higher in households where biomass cooking takes place indoors. As a result of these possibilities, the present study assessed simultaneously a range of lifestyle factors; these consisted of cooking methodology (type of cooking fuel and place of cooking), hand-washing and smoking by parents.

Several mechanisms have been proposed for plausibly linking in a biological context, household air pollution, and ARI among children; the most widely accepted of these are links related to cooking with a biomass fuel and to cigarette smoking in the household; these were recently systematically reviewed by Gordon et al. [15]. In addition to this review, a longitudinal study in Kenya has measured the average daily exposure to particulate matter (e.g., PM_10_) emitted by biomass combustion; the findings revealed that exposed children who were under 5 years old had a higher risk of ARI as compared to their older counterparts [16]. In another study, Sonego et al. conducted a systematic investigation in low and middle income countries studying the link between low socioeconomic status and ARI; their results supported such an association [17]. The findings were also supported by a population based study in Nepal [18]. Another population-based study in Zimbabwe studied the association between household use of biomass fuels and ARI among preschool children and their findings supported the hypothesis that biomass fuel utilization and region of residence were significantly associated with a higher risk of ARI, even after controlling for the children’s gender and birth order [12].

The empirical gaps in the current literature thus seem to involve the relationship between personal lifestyle and related health behaviors, as practiced in households and neighborhoods, and the risk of ARI symptoms [9, 19]. For example, households using biomass as a cooking fuel are clustered in specific communities and the observed community effect of biomass cooking may also involve effects related to lifestyle behaviors within individual households. Thus an increased risk of ARI is therefore not necessarily connected to the community’s collective lifestyle but may rather be associated with individual lifestyle practices within the household [20].

The present study is guided by the social ecological theory [21, 22] which focuses on the complex interplay between the nested environmental levels that envelop children and the individuals in their immediate environment in terms of their influence on health [21]. The theory emphasizes that individuals are embedded within a context and to an extent their lifestyles practices are the result of various societal and social influences; these originate from their immediate environment and include household and community characteristics. Households that are integrated within a given environment are likely to be the most proximal niche in which modifiable daily lifestyle practices can act to shape children’s health [23]. We thus hypothesized that young children with symptoms of ARI will show an association between the presence of ARI and poor personal health-related practice, as well as their risky contextual lifestyle factors.

Furthermore, Amoako et al., who used a population based survey, explored the relationships between the proximate/socio-economic determinants and poor health outcomes among children who have orphan/vulnerable child (OVC) status. They found that OVC status would seem to put the child in peril of ARI; and that the factors that may contribute to the vulnerability of OVC include absence of parental care, a lack of environmental hygiene, their vaccination status, and their ability to access healthcare [24]. In this context, Watts et al. used the Zimbabwe OVC Baseline Survey to investigate similar problems and found that OVC were at greater risk of ARI due to their limited access to medical care including vaccinations [25].

Nonetheless, the above studies have not examined the associations between childhood ARI symptoms, lifestyle factors, personal hygiene in particular, and OVC status in a sub-Saharan African country. Using a national sample of children under five in Nigeria, the main objective of this study is to better understand the association between lifestyle factors and ARI symptoms. Our conceptual framework regards lifestyle factors at both individual and community levels are our main interest and we posit links between lifestyle factors, OVC status, and ARI symptoms. That is, the present study aims to examine whether lifestyle factors, as measured by cooking methodology, household smoking status, and personal hygiene, are associated with the symptoms of ARI, while taking OVC status, as well as other individual and contextual-level social risks, into consideration.

Nigeria, is one of the highest contributor to under-five mortality globally [7] with its under-five mortality (U5M) standing at 109 out of 1000 live births [26]. This is far from achieving the proposal that all countries reduce their U5Ms to as low as 25 out of 1000 live births by 2030 [27]. A progress report in 2015 by the International Vaccine Access Center, revealed that Nigeria is the second largest contributor world-wide to the global burden of child pneumonia and diarrhea; in this context ARI is one of the major causes of U5M [6, 28].

## Methods

### Population

We used cross-sectional data from the 2013 Nigeria Demographic and Health Survey (NDHS), a nationally representative set of data. The survey employed a national probability sample of households that involved a three-stage cluster sampling technique. The country was stratified into 36 states and the Capital Abuja; thus there were 37 districts overall. During the first stage, the primary sample unit (PSU), was based on the 2006 Nigeria population census enumeration areas (EAs). Each PSU corresponds with a smaller geographic unit (such as a neighborhood in an urban area or a village in rural area); this gave rise to a total of 896 clusters. A sample cluster within the PSU was then selected using a probability proportional to population size approach. During the second stage, households were selected within each PSU by systematic sampling of the households present in each selected cluster. The third stage involved the distribution of households across each state, which followed a probability proportional to the urban and rural areas within Nigeria; the final results consisted of a total of 40,680 households.

Data collection of the 2013 NDHS was carried out by trained field workers from the 36 states in Nigeria. The questionnaires was translated into the three major Nigerian languages, Hausa, Igbo, and Yoruba; these were then pre-tested, re-fined, and finalized for the survey and finally back translated to ensure that the questions measured what they were intended to measure. The questionnaire were filled-in by the interviewers in order to collect the required information [29].

Among the successfully sampled households, information on 31,482 children (a combination of dead/alive children) under the age of 5 years was collected with a 95.2 % response rate. In this study, we defined a community as a sample cluster (that is a PSU), which usually was a village or an urban census block. In addition, we restricted our analyses to children who were under 5 years of age and were alive at the time of sampling. The final sample consisted of 28,596 pre-school children and this large sample size has enough statistical power for multi-level analysis. We compared the characteristics of the children included in the study with those of the children who were excluded. A total of 2886 children were excluded due to either survival status or unavailability of information on ARI symptoms. The children excluded were more likely to be older, to be socioeconomic disadvantaged and/or to reside in North western regions (the results not tabled). The dataset is available online from ICF international, Rockville, MD, USA [30]. The study protocol used secondary data analysis of the DHS and was approved by the Research Ethics Committee of National Yang-Ming University (Taipei, Taiwan).

### Outcome and major explanatory measures

The outcome of the study was symptoms of ARI that was defined and measured by the 2013 NDHS [30], an women’s health questionnaire that was administered to eligible women (15–49 years) in order to reveal information on the respiratory health of children aged 0–59 months. Mothers were asked whether their children under 5 years old had been ill with a cough during the 2 weeks preceding the survey. For children who had a cough, the mother was additionally asked if the child, when sick with a cough during the last 2 weeks prior to the survey, also suffered from short, rapid breathing accompanied by a fever. Children who met all of the abovementioned criteria were regarded as having ARI and were coded with a value of 1; otherwise, the children were coded with a value of 0.

Lifestyle factors questions derived from the household data were made up of characteristics related to the individual’s style of living as observed by the interviewers. They consisted of assessments of the cooking method used in the household [13, 14], the smoking status of members of the household [14] and the personal hygiene practices of the household [11]. Household cooking method was derived from two variables, namely the major type of fuel used for cooking by a given household and the place where cooking was conducted in a given household.

The cooking methods were grouped into five categories: households using kerosene/charcoal outdoors or in a separate place, households using biomass cooking indoors, households using biomass cooking outdoor and in a separate place, households using kerosene/charcoal in the house and various other cooking systems that are less polluting cooking methods than the above, such as gas or electricity. The smoking status of any member of the household was asked in a non-specific manner in the household data and this information was classified into whether or not a household member smoked. The interviewers observed child hand washing and this practice was dichotomized into either observed or not within the household.

In addition to the above, also from the household data, the identification of *orphans and vulnerable children (OVC)* was carried out by identifying individuals who had experienced the death of a family member, who had a parent who had been ill for at least 3 months in the past 12 months, or who came from a household where a member of that household member had been ill for at least 3 months in the past 12 months [31]. We used a set of variables from the survey, such as whether or not a child had one or both parents dead, whether the child had lived with a parent who had been sick for at least 3 months in the past 12 months, and whether the child had lived in a household where at least one adult has been sick for at least 3 months or even died in the past 12 months. If a child had one or more of such experiences, the child was coded as 1, otherwise the child was coded as 0.

Based on prior studies [13, 18, 32], we also included several individual control variables that are related to the socio-demographic background of the children, namely gender, age, and birth order, as well as the socio-demographic background of the children’s mothers, namely maternal education and household wealth index. These covariates have often been tied to the symptoms of ARI.

Table 1 gives the categorization and distribution of community control variables from the 2014 DHS dataset in Nigeria. We have included five community-level measures: community wealth index, community OVC status, community use of biomass cooking fuel, place of residence and the region of the province. The above variables are classified into two categories, derived and integral, in order to establish the characteristics of each community [33]. The former were constructed by aggregating the household or individual survey data at sample cluster level, that is the PSU. Community wealth index, community OVC status, and community use of biomass cooking fuel were constructed from an aggregate of households within a given PSU with further grouping into quartiles or tertiles. The computation of the integral community variables was done by extraction from the census of the population and the housing; these resulted in a place of residence, namely urban or rural [34] and a province of residence, namely North West, North Central, North East, South East, South South, and South West [35].

**Table 1:** Hypothesized neighborhood socioeconomic influences, together with the distribution and categorization of neighborhood-level variables, which were used to predict the likelihood of development of ARI symptoms; 2013 NDHS (children 0–5, *n* = 896)

Neighborhood characteristic		Percentage or mean (Std Dev)	Categorization
Community use of biomass cooking fuel		78.82 (40.85)	Community biomass use: 0–1
	Low (0 to 0.738)	27.87 (44.84)	Lowest use of biomass cooking fuel
	Median (0.739 to0. 98)	90.57 (29.22)	Median use of biomass cooking fuel
	High (0.981 to 1)	99.84 (3.87)	Highest use of biomass cooking fuel
Community wealth index		44.55 (49.70)	Lowest quartile versus higher quartile
	The first quartile (0 to 0)	0	
	The second quartile (2 to 40)	16.31 (36.95)	
	The third quartile (40 to 92)	70.18 (45.74)	
	The fourth quartile (93 to 100)	98.82 (10.77)	Highest proportion of middle, rich and richest in the community
Community OVC status		4.23 (20.13)	Proportion of OVC within a community: 0–1
	Low (0)	0	Lowest OVC rate in community
	Median (1.5 to 5.0)	3.31 (1)	Median OVC rate in community
	High (5.1 to 50)	11.02 (5.5)	Highest OVC rate in community
Residence			
	Urban	35.88 (1.10)	
	Rural	64.11 (1.10)	
Province			
	North West	17.27 (0.89)	
	North Central	13.71 (0.84)	
	North East	36.60 (1.06)	
	South East	8.81 (0.67)	
	South South	9.46 (0.54)	
	South West	14.13 (0.83)	

### Analytical strategy

We began the various analyses by characterizing the distribution of communities, the distribution of households, the distribution of children-level characteristics, and the prevalence of ARI symptoms by sample characteristics. Due to the hierarchical structure of DHS data, children nested within a community are often exposed to a common set of community derived influences. Children from the same community often practiced lifestyle behaviors that more similar to each other than to those of children from other communities. We thus employed for the analysis Stata 13.0; this has been updated with the Gllamm program for random intercept multilevel models [36]. In the modeling strategy, children at level 1 were nested within communities at level 2. The random effect at the community (PSU) level was found to be significant (*p* < 0.05) with an intra-class correlation coefficient of 0.29, demonstrating that almost 30% of the total variation with respect to having ARI symptoms was at child’s community.

We elaborated on the significance of individual lifestyle and health behaviors by progressively adjusting for individual, household and community characteristics. The first elaboration targeted lifestyle and health behaviors in order to investigate the association between various preventive practices and ARI symptoms (Model 1), after adjusting for child’s OVC status, child’s gender, child’s age, birth order, maternal education and household wealth. Next, we added community related characteristics (e.g., community OVC, region, urban residence, community wealth, and community use of cooking fuel) to the model in order to test for possibility that confounding factors associated with lifestyle and health behaviors might affect any relationship with ARI symptoms (Model 2). All the steps in the analysis took into account the multi-stage sampling design of the study and adjusted for other confounding variables, such as related individual and household characteristics.

## Results

**Fig. 1:**
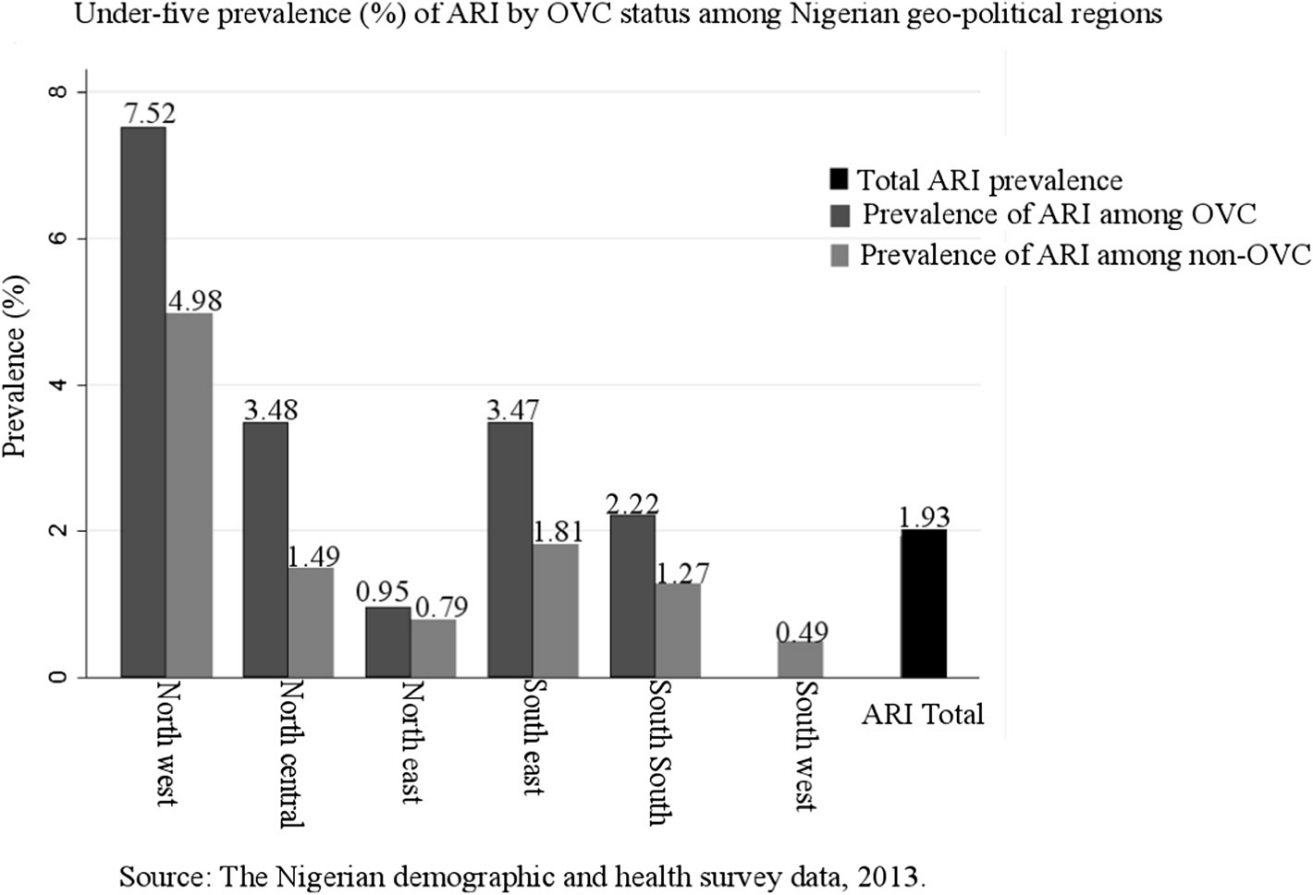
Prevalence of acute respiratory tract infection (ARI) symptoms among individuals with the status of orphans/vulnerable children (OVC) by region

Table 2 shows the distribution of household’s lifestyle behaviors and children characteristics. About two-fifths (42.11 %) of households used biomass fuel for indoor cooking, while less than one-tenth (6.82 %) of households had a member that smoked. Finally, about two-fifths (61.22 %) of young children did not practice hand washing. Overall the ARI prevalence was 1.93 %. A higher prevalence of ARI symptoms was found among children living in a household with in-house biomass cooking (2.07 %), among children living in a household where a household member smoked (2.89 %); and among children who did not practice hand-washing (2.27 %). In addition, compared with non-OVC, OVC had a much higher prevalence of ARI symptoms (3.54 %). The prevalence of ARI symptoms by OVC status and region further indicated that OVC who lived in the North East region (7.52 %) were specifically at a much greater risk of ARI symptoms (Fig. 1).

**Table 2:** ARI prevalence by sample characteristics for children under 5 years of age [percentage or mean (Std Dev)], NDHS 2013

		Total (*N* =28,596)		ARI prevalence
		Percentage or mean	Std. Dev.	Percentage or mean
*Explanatory variables*				
Lifestyle and health behaviors				
Cooking method				
	Kerosene/charcoal outdoors or in a separate place	10.03		0.64
	In-house use of biomass fuel for cooking	42.11		2.07
	In-house use of kerosene/charcoal for cooking	11.33		1.20
	Cooking using biomass fuel outdoors or in a separate place	34.86		2.39
	Other	1.68		1.29
Smoking status of members of the household				
	Yes	6.82		2.89
	No	93.18		1.86
Hand-washing				
	Observed	38.78		1.36
	Not observed	61.22		2.27
Individual and household covariates				
*Child’s background*				
Gender				
	Female	49.89		1.96
	Male	50.11		1.90
Age (in months)		28.14	17.32	
	0–5	10.32		1.46
	6–11	11.27		2.82
	12–23	20.37		3.08
	24–35	18.95		2.08
	36–59	39.08		1.12
Birth order		3.88	2.54	
	1–3	52.20		1.79
	4–6	31.73		1.76
	>6	16.07		2.70
OVC status				
	OVC	3.91		3.54
	Non-OVC	96.09		1.86
*Household’s background*				
Maternal education				
	No education	48.31		1.96
	Primary education	19.16		2.05
	Secondary and above	32.53		1.81
Household wealth				
	Richest	18.14		0.98
	Richer	18.14		1.35
	Middle	19.07		2.03
	Poorer	22.40		2.88
	Poorest	22.96		2.09
Outcome measure				
Symptoms of ARI				1.93

^*a*^Unweighted N’s and weighted percentages and means are reported. Percentages may not add up to 100 due to rounding

Table 3 presents the multilevel logistic regression models used to investigate the relationship between lifestyle and health behaviors in relation to ARI symptoms. Model 1 shows that an unhealthy lifestyle and unhealthy behaviors are significantly associated with ARI symptoms. The odds of having ARI symptoms were increased among young children who lived in households using biomass fuel for cooking indoors (AOR = 2.38; *p* < 0.01), who lived in households using in-house kerosene/charcoal for cooking (AOR = 2.08; *p* < 0.01), who lived in households using biomass fuel for cooking outdoors (AOR = 2.54; *p* < 0.01), who lived with a member of the household who smoked (AOR = 1.37; *p* < 0.05), and who lived in households where hand-washing was not observed (AOR = 1.59; *p* < 0.001), even when the analysis was controlled for a wide range of individual and household characteristics.

**Table 3:** Results of the multilevel regressions of the odds of ARI symptoms among young children, 2013 NDHS (*N* = 28,596)

		MODEL 1		MODEL 2	
*Covariates*		AOR	95 % CI	AOR	95 % CI
Lifestyle and health behaviors					
Cooking method (ref = Kerosene/charcoal outdoors or a separate place)					
	In-house use of biomass fuel	2.38^**^	1.34–4.24	2.30^**^	1.26–4.20
	In-house use of kerosene/charcoal	2.08^*^	1.17–3.70	2.11^**^	1.19–3.72
	Outdoor or separate use of biomass fuel	2.54^**^	1.45–4.45	2.28^**^	1.27–4.10
	Other	2.51	0.95–6.64	2.42	0.94–6.25
Smoking of members of the household (ref = No)					
	Ever smoked	1.37^*^	1.01–1.87	1.33	0.98–1.81
Hand-washing (ref = Observed)					
	Not observed	1.59^***^	1.28–1.98	1.66^**^	1.33–2.07
Neighborhood characteristic					
Community OVC status (ref = Low)					
	Median			1.28	0.94–1.75
	High			1.74^***^	1.34–2.25
Region of residence (ref = North West)					
	North Central			0.39^***^	0.27–0.57
	North East			0.15^***^	0.11–0.20
	South East			0.30^***^	0.19–0.48
	South South			0.35^***^	0.22–0.57
	South West			0.26^***^	0.15–0.43
Community wealth index (ref = The first quartile)					
	The second quartile			1.03	0.67–1.58
	The third quartile			0.94	0.54–1.62
	The fourth quartile			1.05	0.56–1.95
Community use of biomass cooking fuel (ref = Low use)					
	Median use			0.82	0.51–1.31
	High use			1.07	0.63–1.81
Urban residence (ref = Rural)				0.95	0.68–1.33
Individual and household backgrounds					
Female gendered (ref = Male)		1.02	0.85–1.21	1.03	0.86–1.22
Birth order (ref = 1–3)					
	4–6	0.92	0.75–1.13	0.94	0.76–1.15
	>6	1.35^*^	1.07–1.71	1.37^**^	1.08–1.73
Age in months (ref = 36–59)					
	0–5	1.21	0.85–1.73	1.20	0.85–1.71
	6–11	2.67^***^	2.02–3.53	2.66^***^	2.01–3.51
	12–23	2.88^***^	2.28–3.64	2.85^***^	2.25–3.60
	24–35	1.82^***^	1.40–2.36	1.80^***^	1.30–2.34
OVC (ref = Non-OVC)		1.43^*^	1.01–2.02	1.27	0.90–1.80
Maternal education (ref = Secondary or above)					
	No education	0.63^**^	0.47–0.84	0.63^**^	0.47–0.85
	Primary education	0.70^*^	0.53–0.93	0.69^**^	0.53–0.92
Household wealth (ref = Richest)					
	Richer	1.19	0.76–1.87	1.15	0.74–1.79
	Middle	1.72^*^	1.06–2.82	1.61	0.97–2.65
	Poorer	2.72^***^	1.64–4.51	2.39^**^	1.38–4.14
	Poorest	1.94^*^	1.13–3.32	1.60	0.88–2.91
*Model statistics*		Coeff	SE	Coeff	SE
Log likelihood		−2561.92		−2479.75	
Comparison to previous model					
	Chi-square			82.17^***^	
	Degrees of freedom			13	
Random variance					
Intra-class correlation (ICC)		0.26^*^		0.13^*^	
Variance between neighborhoods		1.17^***^	0.01	0.70^***^	0.08

Intra-class correlation (ICC) measures the degrees of clustering with random intercepts. The correlation of the 2-level multilevel logistic regressions is calculated by σ_μ_^2^/ [σ_μ_^2^ + π^2^/3], where σ_μ_^2^ denotes neighborhood- level variance

*Abbreviations*: *AOR* represents adjusted odds ratios for sample cluster, *CI* represents confidence interval

^*^ *p* < 0.05; ^**^ *p* < 0.01; ^***^ *p* < 0.001

Model 2 added the neighborhood characteristics. Compared to Model 1, their inclusion produced some reductions in the significance of the coefficients with respect to lifestyle; these consisted of household cooking method and household smoking status. These findings indicate that some lifestyle effects are somehow redundant with respect to neighborhood characteristics. Nevertheless hand-washing still had an independent and significant association with ARI symptoms. Turning to the neighborhood level, there were some significant effects of the neighborhood-level characteristics on the prevalence of ARI symptoms. Young children living in a community with a high proportion of OVC were more likely to have ARI symptoms (AOR = 1.74, *p* < 0.001). Furthermore, the OR of having ARI symptoms was higher for children living in the North West region. However, community education and community wealth were found not to be significant when trying to explain ARI symptoms. Finally, no significant differences were found in relation to urban versus rural residence.

Model 2 also revealed a number of statistically significant individual background effects. The ORs for ARI symptoms were significantly higher for children living in households who were poorer compared with those living in the richest households. Surprisingly, compared to mothers with secondary or higher levels of education, a lower odds of having ARI symptoms were found among mothers with no education (AOR = 0.63; *p* < 0.01) and primary education (AOR = 0.69; *p* < 0.01). In addition, we did not find a statistically significant association between ARI symptoms and a number of other individual characteristics such as gender and OVC status.

The multilevel models were able to assess the relationship between variation in lifestyle factors and the odds of having ARI symptoms. Using log likelihood statistics as a means to assess the model-fitting, Model 2 showed a significant improvement in fit over Model 1 [*χ*^2^ (13) = 82.17; *p* < 0.001], as shown at the bottom of Table 3 [37]. Furthermore, a significance decrease in neighborhood variance from 1.17 in Model 1 to 0.70 in Model 2 was observed. This means that about 40 % of ARI variance is attributable to neighborhood and other individual factors. These findings reveal a plausible contextual phenomenon that would seem to help shape the community differences among young children regarding having ARI symptoms.

## Discussion

Using multilevel modeling to disentangle the dynamics of interplay occurring between each environmental niche, this study addressed gaps in the literature regarding lifestyle and health behaviors and their relationship with ARI symptoms, as well as further assessing the effect of neighborhood and individual characteristics on such relationships by including lifestyle factors at various levels, which in turn attributes the unexplained variation in ARI symptoms to different levels [38]. In the analyses, personal lifestyle factors showed a significant association with the children’s ARI symptoms. Young children living in households that used biomass fuel for cooking fuel indoor, or used biomass fuel for cooking fuel outdoor were more than two times more likely to have ARI symptoms than those living in households that use either kerosene/charcoal for cooking outdoors or where cooking was conducted in a separate place [16, 39, 40]. Children who practiced hand-washing had a 34 % lower odds of having ARI symptoms [11]. Nevertheless, the significant association found for the smoking status of household members regarding children’s ARI symptoms was reduced to non-significance after the inclusion of neighborhood and individual characteristics. Prior studies have suggested that second-hand smoke exposure in households is a risk factor with respect to ARI symptoms. However, it needs to be noted that Nigeria has one of the lowest levels of smoking prevalence around the world [41, 42] and taking this into consideration, the small proportion of active household smokers who live with children are still likely to expose them to an increased risk of ARI, but this may not have been detectable at the significance level used in this study [43, 44].

Inconsistent with prior studies [20, 34, 45], our findings do not indicate a significant association between contextual lifestyle factors at neighborhood level and the prevalence of ARI symptoms. This lack of significance at the neighborhood level points towards the possibility that the proximal lifestyle practiced within the household of a given likelihood has more relevance to the disease than any neighborhood effect. For instance, our findings do not show a significant association between communities that have a higher rate of cooking with biomass fuel indoors and children’s ARI symptoms. In a separate analysis not shown here, we found that the use by communities of biomass cooking fuels was prevalent in Nigeria irrespective of ARI status. This lack of variation may help to partially explain why these findings did not reach significance [20]. The homogeneity in exposure to biomass combustion in the place of residence is extensive.

Guided by social ecological theory [21, 22], we proposed that several community characteristics, such as proportion of OVC within a community and the region of a community, can also be associated with ARI symptoms among young children showing an interplay between each environmental niche and child developing ARI symptoms, such as the proximal effect of the household level environment and lifestyles, specifically, where children are nested. This includes how it is determined by community level factors or resources, which intuitively are likely to interact with household lifestyles and behaviors. Our findings support our hypothesis that communities with a high OVC rate have significantly higher odds of having ARI symptoms among young children. In addition, the region of residence can also be another indicator of community characteristics. The North Eastern and North Western regions of Nigeria are exposed to higher levels of dust exposure; this is because these areas of Nigeria lie along the Gulf of Guinea trajectory. Specifically, dust and sand storms are not uncommon in these regions, namely across northern Nigeria [46]. In addition, homes situated on the road side exposed to road traffic pollution [47] and there also may be poor indoor ventilation of houses [48]. Furthermore, the northern Nigeria has suffered from serious political problems and religious unrest in recent times, which has resulted in within region migration [42]; it is within the regions affected by these factors that we have found young children are at higher risk of ARI symptoms. Yet, another plausible explanation for this variation in ARI symptoms at the regional level across Nigeria may be the presence of social and economic development spatial inequality between different areas [49].

Our analysis supports the findings of various prior studies [18], namely that the most vulnerable age for having ARI symptoms is between 12 months old and 23 months old. Overcrowding is another major reason for ARI symptoms as our results found that children with a higher birth order were also more likely to ARI symptoms. Yet, our analysis indicated a significant negative association between maternal education and ARI symptoms. This suggests that children born to uneducated mothers were less likely to develop ARI symptoms. This is in line with a pilot study from Indonesia, which found that a mother’s education level had an indirect effect on childhood pneumonia and respiratory illness [50]. It seems likely that educated mothers are more likely to be autonomous and thus are also more likely to be employed; this will result in their children being left alone or in care of someone whose lifestyle may influence or exacerbate the likely of symptoms of ARI. Another possibility is an underestimation of the occurrence of ARI symptoms due to recall bias of the event, ARI symptoms may not have been fully understood by uneducated mothers, or because the children of uneducated mothers have a higher likelihood of dying from ARI and thus being excluded from this study [13]. Finally, our findings did not find a gender difference regarding ARI symptoms in Nigeria and this is also consistent with prior findings [12].

The present study has used advances in multi-level modeling strategies in order to avoid the possibility of the bias that is often associated with sample clustering. Yet, our findings need to be interpreted within the context of the study’s limitations. In addition to the common limitations associated with self-reported measures of lifestyle factors, the association of individual and neighborhood factors with ARI symptoms might be influenced by endogeneity problems. Due to the cross-sectional nature of the data, this limits our ability to infer a causal relationship. In addition, having found that children living in poorer households using biomass fuel clearly have a high risk of dying from ARI, this leads to a possibility of some selection in the cross-sectional sample used in this study; this in turn leads to the possibility of a downward bias on the effect of cooking smoke on ARI symptoms. However, in the light of a high prevalence of ARI, together with the relatively small number of deaths in the sample, the effect of this bias on our estimated effect is likely to be minimal.

In addition, recall bias and underreporting of ARI symptoms is also a possible issue among those living in households using biomass fuel, the majority of whom are individuals who are most likely to lack awareness of ARI symptoms during the 2-week period used in this study. This self-report measure thus may contribute to an underestimation of ARI symptoms as the information on ARI was not validated by a medical examination. In situation of a developing country such as Nigeria, where clinical information on ARI is usually not available or is relatively unreliable, the measure of ARI symptoms used in our study has been shown to provide an accurate estimation of ARI. On average, within a population; this is because the definition is based on a combination of three symptoms that are easily recognizable by mothers, namely fever, cough and rapid breath. Such an approach also has the advantage of minimizing misclassification [12]. It should be noted that due to data limitation, seasonal factors cannot directly be taken into account. However, prior research has validated ARI measures in DHS surveys by using the month of interview as a proxy season factor. Evidence shows no significant influence on the findings with and without this season factor [51]. Finally, our analyses are based on using sets of multilevel logistic models with random intercept and fixed coefficients only. Our findings cannot provide evidence of the effects of individual factors variance across neighborhoods. However, by using a multilevel analytical approach, our study does provide important insights and does identify lifestyle and health behaviors from a multilevel perspective in Nigeria, which is a country with a serious ARI epidemic. Future research is needed to assess the potential community differences in lifestyle and the longitudinal effect of this dynamic variable on ARI symptoms among young children in sub-Saharan Africa.

## Conclusions

Despite the above caveats, the results from the present study fill several noteworthy gaps in the current literature. The large sample size of the Nigerian DHS survey permits extensive analyses of subgroups and their lifestyle and community differences; additionally, the unique design and sampling procedures provide an important opportunity for assessing the association between individual/household lifestyle characteristics, community influences and ARI symptoms. Much of the existing literature related to ARI symptoms among young children in African countries is descriptive and has been focused at the individual level. This study extends these efforts by examining neighborhood mechanisms through which differences in lifestyle and health behaviors and differences in neighborhood environment are associated with ARI symptoms. Furthermore, the DHS survey provides a wealth of information about several key constructs, namely lifestyle factors, OVC status, and neighborhood resources; this information allows an examination of patterns related to ARI symptoms among young children in much greater detail than has been possible in prior research. The findings on the lifestyle/health variables, and their relationships to ARI symptoms within this context, have thus been able to advance our knowledge. This will allow researchers and policy makers to evaluate the potential effectiveness of current programs that are aimed at improving public policy regarding ARI among young children.

## Abbreviations

ARI: Acute respiratory infection
EA: Enumeration area
OVC: Orphan and vulnerable child
PSU: Primary sampling unit
U5M: Under five mortality
